# Evaluation of Anticancer Activity of Fruit and Leave Extracts from Virus Infected and Healthy Cultivars of *Vitis vinifera*

**Published:** 2013-07-02

**Authors:** Zahra Esfahanian, Mandana Behbahani, Mehrnaz Shanehsaz, Mohammad Javad Hessami, Mohammad Ali Nejatian

**Affiliations:** 1 Department of Biotechnology, Faculty of Advanced Sciences and Technologies, University of Isfahan, Isfahan, Iran; 2 Agricultural Research, Education and Extension Organization. Quazvin, Iran

**Keywords:** *Vitis vinifera*, Anticancer, Virus

## Abstract

**Objective:**

Grape virus diseases are a serious problem in Iran. Leaves and fruits of grape have been used for different purposes like cooking in Iran. The present investigation was carried out to study on the cytotoxic-activities of extracts of fruits and leaves of *Vitis vinifera* from both virus-free and virus-infected grape cultivars against breast cancer cell line (MDAMB- 231) and human embryonic kidney normal cell line (HEK 293).

**Materials and Methods:**

In this experimental study, the considered grape cultivars were as follows: Rish Baba Sefid, Shahani Ghasre Shirin, Rotabi Zarghan, Asgari Najaf Abad, Fars, Kaj Angor Bojnord, Sarkesh Shiraz and Siahe Zarqan. A real-time multiplex polymerase chain reaction (real-time Multiplex PCR) assay was applied to detect virus infected cultivars. The cytotoxic effect of the methanol extracts of different *Vitis vinifera* varieties on cultured cells was monitored using (3- (4, 5-Dimethylthiazol-2-yl) -2, 5-diphenyltetrazolium bromide (MTT) assay at different concentrations (62.5, 125, 250, 500, 750, 1000 μg mL^-1^).

**Results:**

Among these cultivars, *Grapevine fanleaf virus* (GFLV) along with related symptoms was detected in Siahe Zarqan and Fars. Methanolic extracts of leaves and fruits of *Vitis vinifera* from both virus free and virus infected cultivars showed a range of limited to moderate cytotoxic activity. However, methanol extract of leaves belonged to virus infected cultivars was found to have strong cytotoxic effect against MDA-MB-231 at different concentrations.

**Conclusion:**

*Grapevine fanleaf virus* (GFLV) can potentially increase the cytotoxicity of grape cultivars.

## Introduction

*Vitis vinifera*, a species of grape, is native to the Mediterranean region, central Europe and Southwestern Asia. It has been planted all over the world and is used for both medicinal and nutritional value. Previous studies on some grape varieties have shown that most of the cultivars possess medicinal properties, such as anti-inflammatory and anti-cancer effects (1, 2).

Through extracting natural compounds from fruits and leaves of *Vitis vinifera* in recent decades, a number of phenolic compounds, such as gallic acid, catechin, resveratrol and a wide variety of procyanidins have been isolated and studied for their biological activities and health-promoting benefits (3). Phenolic substances are synthesized during the process of plant growth, whereas the presence of some stress factors, like ultraviolet radiation and disease also increase the synthesis of them (4). Grape virus diseases are a serious problem in Iran. More than 55 viruses or strains classified in 20 different genera are known to infect grapevine crops worldwide (5), and substantially reduce yield and quality (6). The oldest known virus disease of *V. vinifera*, generated by *Grapevine fanleaf virus* (GFLV), causes poor berry set and severe yield losses, even in some varieties, yield loss exceed 80% of entire grape production (7). GFLV infects almost all *Vitis* species, and there have been several reports of this type of infection among Iranian grape cultivars (8). The cytotoxic activities of *V. vinifera* cultivars have been investigated against different cancer cell lines (HL- 60, MCF-7, HT-29 and HeLa) (9, 10); however, no scientific study is being presented yet about anti-cancer activities of the virus infected cultivars. Since grapevine viruses affect most varieties in Iran, it is of utmost importance to identify the cytotoxic activities of these grape varieties infected by virus. Furthermore, the anti cancer properties of Iranian grape cultivars have not also been investigated. Therefore, the present investigation was carried out to study on the cytotoxic activities of extracts of fruits and leaves of *Vitis vinifera* from both virus-free and virus-infected grape cultivars against breast cancer cell line (MDA-MB-231) and human embryonic kidney normal cell line (HEK 293).

## Materials and Methods

### Plant material

In this experimental study, our first group included virus free leaves (samples) of six plants for each of eight varieties of *Vitis vinifera*e, namely Rish Baba Sefid, Shahani Ghasre Shirin, Rotabi Zarghan, Asgari Najaf Abad, Fars, Kaj Angor Bojnord, Sarkesh Shiraz and Siahe Zarqan, while our second group consisted of virus infected leaves (samples) of six plants for only two varieties of *Vitis vinifera*e, including Siahe Zarqan and Fars. All plants were obtained from Qazvin Agriculture and Natural Resources Research Center, Qazvin, Iran, in August 2009. The samples were immediately put on ice for transport, and then stored at -80˚C until the viral RNA of infected leaves was extracted. In order to apply cytotoxicity assay, young and old leaves and fruits from both virus free and virus infected cultivars were also collected.

### Preparation of the plant extract

The grape seed and peel were separated from the grape pulp. The plant materials were carefully dried and powdered. The dried plant samples (30 g) were placed in a stopped conical flask and macerated with 500 mL of 98% (v/v) methanol (Merck, Germany) at room temperature (25-28˚C) for three days with occasional stirring. Each experiment was performed in triplicate (n=3). The solvent was then filtered and evaporated in a vacuum rotary evaporator (Stroglass, Italy) at 45˚C. The residue was placed in the freezedrier (Zirbus, Germany) until dried. The crude extract was stored in a well-closed container, protected from light and kept in a refrigerator at 4˚C. A total of 40 mg of the sample extract were dissolved in one ml of 100% (v/v) dimethyl sulfoxide (DMSO), followed by being sonicated.

### Isolation of total RNA extract

Total RNA was isolated from leaf blades of virusinfected grapes with the method described by Chang et al. with slight modifications (11). All steps were performed at 4˚C. One gram of grape tissues was grounded to a fine powder with liquid nitrogen using mortar and pestle in presence of two mL washing buffer, containing 0.1 mol L^-1^ Tris boric acid (pH=7.4), 0.35 mol L^-1^ sorbitol, 10% PEG 6000 (w/v), and 2% β-mercaptoethanol (v/v). After centrifugation at 15,000×g for five minutes, two mL of the extraction buffer containing 0.1 mol L^-1^ Tris–borate acid (Tris boric acid) (pH=7.4), 1.4 mol L^-1^ NaCl, 0.02 mol L^-1^ ethylendiamin-tetraacetat (EDTA) and 2% cetyltrimethyl ammonium bromide (CTAB) was added, afterward incubated for 20 minutes at 50˚C. Then, 200 μL potassium acetate of five mol. L^-1^, 200 μL ethanol and two mL chloroform were added to the solution. After centrifugation at 15,000×g for 10 minutes, 1/3 volume of 10 mol L^-1^ LiCl and 0.8 volume of isopropylalcohol were added before centrifugation at 15,000×g. The pellet was dried and resuspended in 0.5 mL diethylpyrocarbamate (DEPC)-treated water, then 0.5 mL water-saturated phenol was added. After centrifugation at 15,000×g for 15 minutes, 0.5 mL chloroform/isoamylalcohol was added before centrifugation at 15,000×g. Total RNA was then precipitated over night after addition of 1/3 volume of 10 mol L^-1^ LiCl. Next day, after centrifugation (15,000×g, 30 minutes), the pellet was washed in 75% ethanol and resuspended in DEPC-water. RNA concentration was determined by measuring the absorbance at 260 nm (A260) in a spectrophotometer (Awareness Technology Inc., stat fax 2100).

### Detection of RNA grape viruses by RT-PCR

The mRNA expression patterns of capsid protein genes from Grapevine virus (GVA), Grapevine fleck virus (GFkV), Grapevine Grapevine leafroll-associated virus 3 (GLRaV-3) and RNA dependent RNA polymerase (RdRp) gene of *Grapevine fanleaf virus* (GFLV) were examined by reverse transcription polymerase chain reaction (RT-PCR). RNA extracted from virus-infected plants served as an substrate for reverse transcription and multiplex polymerase chain reaction (Multiplex PCR) using four primer-sets, published previously by Goszczynski and Jooste (8) and Gambino et al. (12) (Table 1). For Real Time PCR, first-strand cDNA was synthesized from five μg of total RNA in a volume of 20 μL of a solution containing four μl reaction buffer (5X), one mmol L^-1^ dNTP, 20 units of RNase inhibitor, five units of AMV reverse transcriptase, and 100 pmol of random hexamer for 45 minutes at 42˚C, followed by 10 minutes at 95˚C. Then, five μg of the first-strand solution was used for PCR reaction in a total volume of 50 μl with 20 mmol L^-1^ Tris-HCl (pH=8.3), 100 mmol L^-1^ KCl, two mmol L^-1^ dNTP, five units of Taq DNA polymerase (Roch, Germany), 2.5 mmol L^-1^ MgCl_2_, as well as 10 pmol of each gene-specific amplification primer. PCR amplification consisted of initial denaturation at 94˚C for 10 minutes, followed by 45 cycles of denaturizing at 94˚C for five seconds, annealing at 54˚C for one minute, extension at 72˚C for one minute, and with a final extension at 72˚C for 10 minutes in a Corbett Research CG1-96 thermal cycler. Four types of viruses, including GVA, GFkV, GLRaV-3 and GFLV, obtained from the Qazvin Agriculture and Natural Resources Research Center, were prepared and were used as positive control. Negative control was tested with only four primer pairs to verify the results. PCR products were analyzed by electrophoresis on a 1.5% agarose gel in 1× tris-borate-EDTA (TBE) buffer, stained with 10 μg m L^-1^ ethidium bromide, and photographed over a UV transilluminator (Stratagene, Heidelberg, Germany).

**Table 1:** DNA primers were employed for reverse transcription-polymerase chain reaction (RT-PCR) amplification of grapevine viruses and for control mRNA sequenc


Gene	Product size (bp)	Location	Sequences 5′-3′	Target Primer

**GFLV**	f-TGCTGGATATCGTGACCCTGT	5506-5527	118	RNA dependent
	r-AAGGTATGCCTGCTTCAGTGG	5602-5623		RNA polymerase
**GFkV**	f-TGACCAGCCTGCTGTCTCTA	6453-6472	179	Coat protein
	r-TGGACAGGGAGGTGTAGGAG	6612-6631		
**GVA**	f-AGGTAGATATAGTAGGACCTA	6591-6612	272	Coat protein
	r-TCGAACATAACCTGTGGCTC	6843-6862		
**GLRaV-3**	f-ACGTTAAGGACGGGACACAGG	13383-13404	336	Coat protein
	r-TGCGGCATTAATCTTCATTG	13699-13718		


### Cell lines and culture medium

Human breast cancer cell line (MDA-MB-231 cells) and human embryonic kidney cell line (HEK 293) were purchased from the Cell Bank of Pasteur Institute, Tehran, Iran. MDA-MB-231 and HEK 293 cells were cultured in RPMI, supplemented with 10% (v/v) fetal calf serum (FCS), 100 U mL^-1^ penicillin and 100 mg mL^-1^ streptomycin, 2mM Lglutamine and 1mM sodium pyruvate. All reagents were purchased from Gibco, Germany.

### Cytotoxicity assay

A total of five extracts from healthy cultivars were obtained, representing five different parts of the plant, like young and old leaf, seed, fruit, skin and pulp extracts of *V. vinifera* in methanol solvent. All extracts were tested under comparable conditions, at different concentrations (62.5, 125, 250, 500, 750, 1000 μg mL^-1^). The cellular cytotoxicity of the methanol extracts from *Vitis vinifera* cultivars on cultured cells was monitored using 3-(4, 5-dimethylthiazol- 2-yl)-2,5-diphenyltetrazolium bromide (MTT) assay (13). The cells were grown in 96-well plates at a density of 5×10^4^ cells per well. After 24 hours, the cells were treated with different concentrations of samples and the incubation was continued for 48 hours. Later, 25 μL of the MTT solution (5 mg/ mL) was added to each well, and the plate was reincubated for four hours. Finally, the medium was removed and 100 μL of DMSO was added in order to solubilize the formed formazan crystals. The amount of formazan crystal was determined by measuring the absorbance at 492 nm using a microplate spectrophotometer (Awareness Technology Inc., stat fax 2100). The relation between surviving fraction and extract concentration was plotted in order to get the survival curve of each cell line after the specified period of time.

### Statistical analysis

Each experiment was carried out in triplicate and repeated two times. The experiments were performed using complete randomized design (CRD), while the obtained results were evaluated using the one way ANOVA. Experiment was a factorial. Statistical analysis was performed using the SAS system, version 6.12. The value of p< 0.05 was considered significant.

## Results

**Fig 1:**
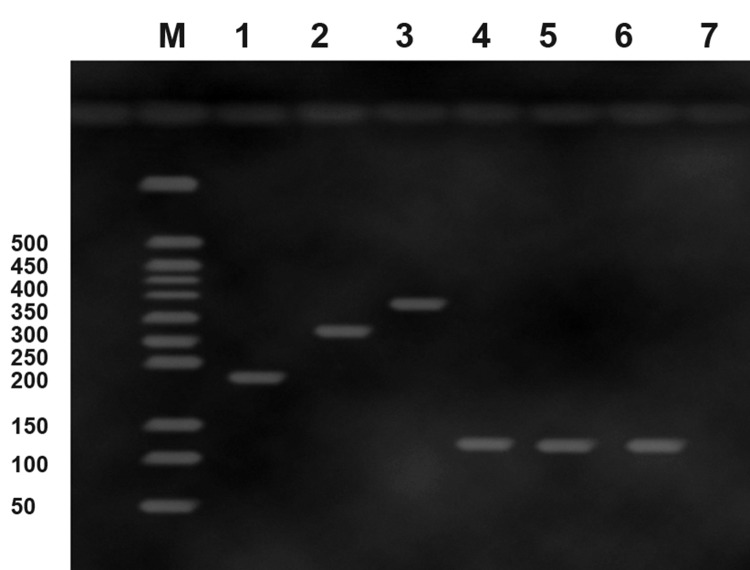
Agarose gel electrophoretic analysis of DNA fragments amplified from infected and healthy grapevines by multiplex reverse transcription-polymerase chain reaction (mRT-PCR). Lane 1, 2, 3 and 4: Positive control for grapevine fleck virus (GFkV; 179 bp), grapevine virus (GVA; 272 bp), grapevine leafroll-associated virus-3 (GLRaV-3; 336 bp), and grapevine fanleaf virus (GFLV; 118 bp), respectively; Lane 5, 6: GFLV infected leaves; Lane 7: Negative control, water with all the primers; and Lane M: 50-bp DNA ladder.

The PCR products are analyzed by gel electrophoresis and compared with the positive controls. Four types of viruses, including GVA, GFkV, GLRaV- 3 and GFLV, obtained from the Qazvin Agriculture and Natural Resources Research Center, were prepared and were used as positive control. Negative control was tested with only four primer pairs to verify the results. The PCR products of positive control, namely GFkV (179 bp), GVA (272 bp), GLRaV-3 (336 bp) and GFLV (118 bp) were analyzed by electrophoresis. Another PCR product with the size of approximately 118 bp was also detected in two grape cultivars (Siahe zarqan and Fars) with viral symptoms on leaves using four primersets (Fig 1). No band for three other viruses (GFkV, GVA and GLRaV-3) was detected in virus infected cultivars. Amplified product of expected sizes was observed only from infected samples, while nothing was observed from healthy plants. However, the different parts of virus free cultivars were tested for cytotoxicity against breast cancer cell line (MDAMB- 231) and human embryonic kidney normal cell line (HEK 293).

**Fig 2:**
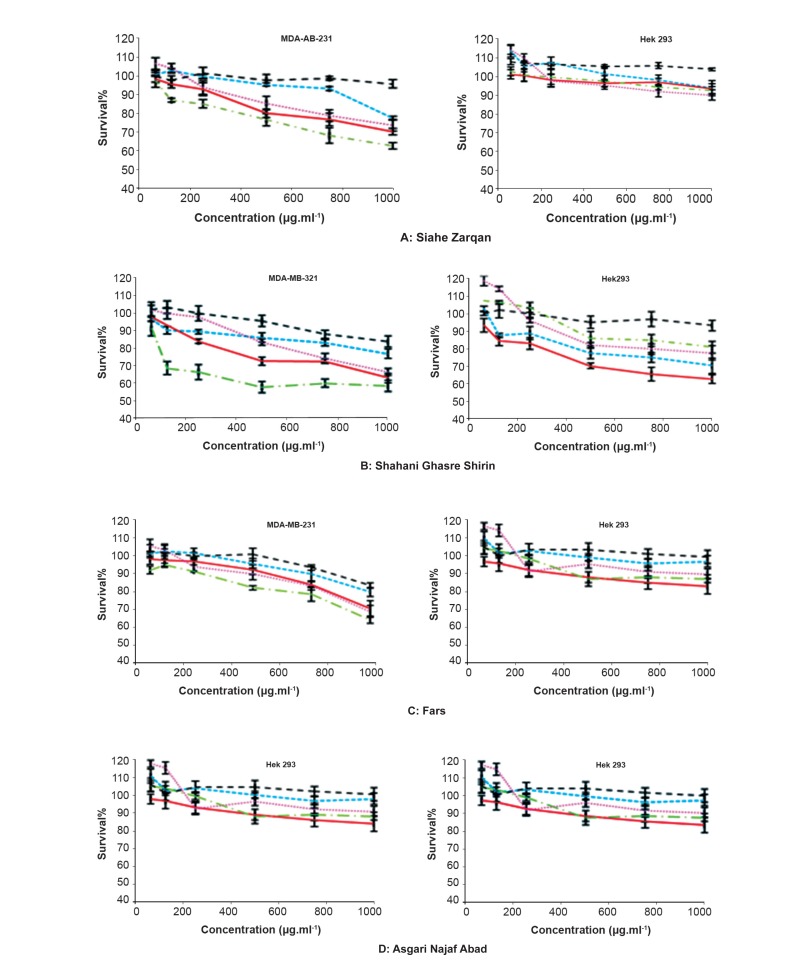
Cytotoxic activity in extracts of young (---) and old leaf ( —— ), seed (……) , skin ( ) and pulp (- - -) of V. vinifera cultivars, including: A. Siahe Zarqan, B. Shahani Ghasre Shirin, C. Fars, and D. Asgari Najafabad.

**Fig 3:**
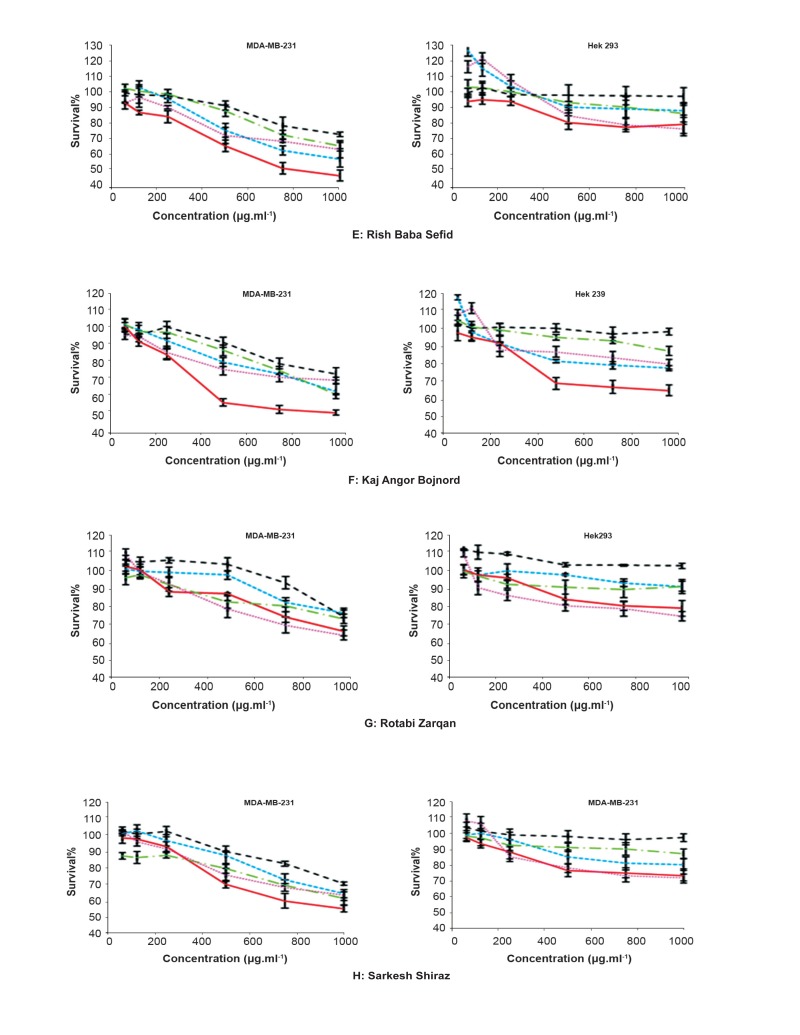
Anti cancer activity in extracts of young (---) and old leaf ( —— ), seed (……) , skin( ) and pulp (- - -) of V. vinifera cultivars, including E. Rish Baba Sefid, F. Kaj Angor Bojnord, G. Rotabi Zarghan, and H. Sarkesh Shiraz.

A total of five extracts from healthy cultivars were obtained, representing five different parts of the plant, like young and old leaves, seed, fruit, skin and pulp extracts of *V. vinifera* in methanol solvent. All extracts were tested under comparable conditions, at different concentrations of 62.5, 125, 250, 500, 750, 1000 μg mL^-1^. All tested extracts exhibited different potency of cytotoxic activities in a concentration-dependent and showed IC50 value greater than 500 μg mL^-1^ against two human cell lines. The methanol on skin extract of all red cultivars (Shahani ghasre shirin, Asgari najaf abad, Fars and Siahe zarqan) showed the most potent activities against MDA-MB-231 cell. The results presented that cytotoxic activity of old leaves of all white and red cultivars were more than young leaves against MDA-MB-231 and HEK 293 cells. The results of cytotoxic activity of different concentration of extracts on cells showed that different methanol extracts of leaf, skin and seed of red and white cultivars were more active against MDA-MB-231 in comparison with HEK 293, while methanol extracts of pulp did not reveal any cytotoxic activity in all different cultivars. Results are represented in figures 2 and 3.

**Fig 4:**
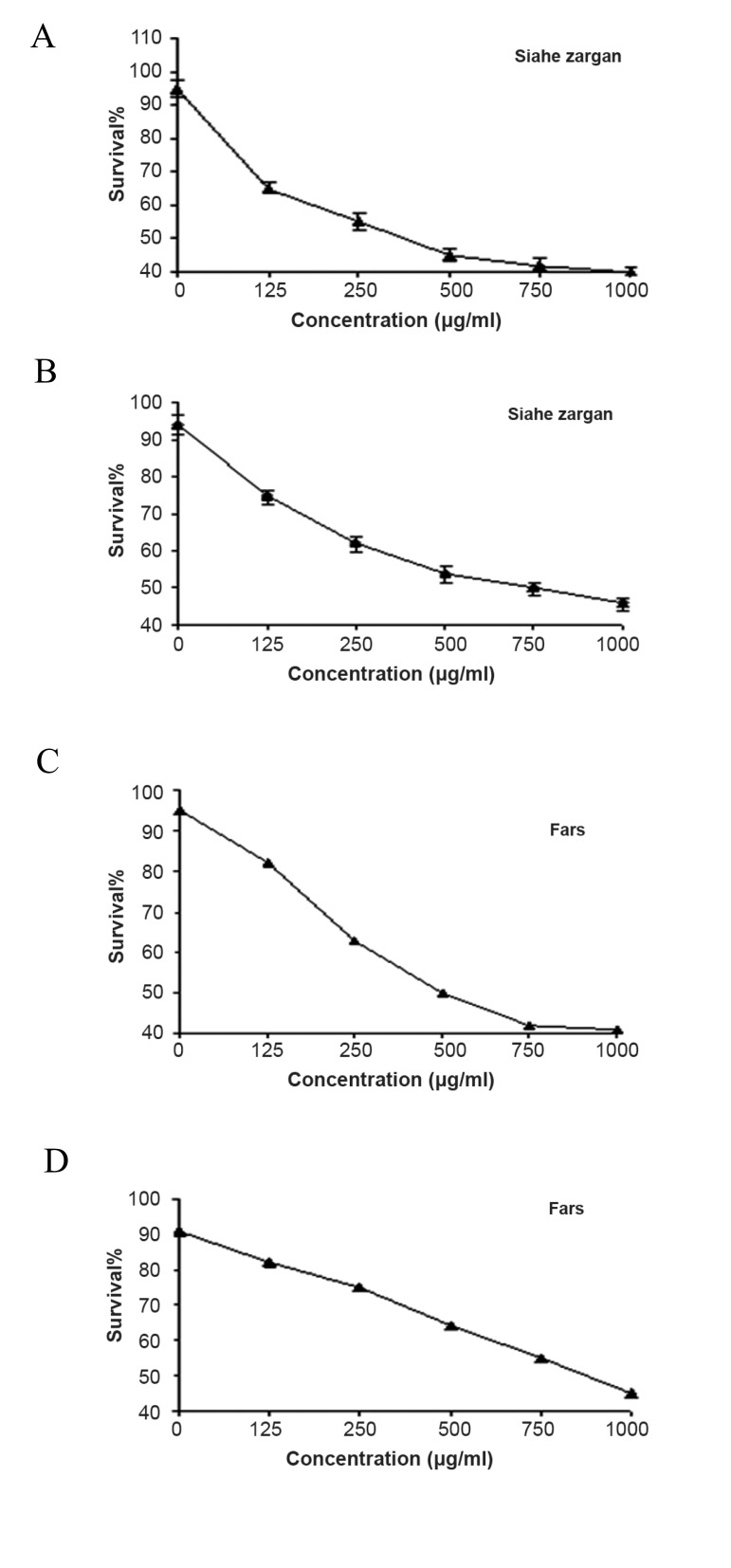
Cytotoxic activity in extracts of old leaf of virus infected (A and C) and healthy (B and D) leaves of V. vinifera cultivars, including Siahe Zarqan (A and B) and Fars (C and D).

The Cytotoxic activity of old leaves extracts of two virus-infected cultivars (Siahe zarqan and Fars) against MDA-MB-231 was measured and compared with virus free cultivars. Both Siahe zarqan and Fars exhibited cytotoxic activities in a dose-dependent with IC50 values of 750 μg/mL and >750 μg/mL in healthy cultivars, while IC50 values of 350 μg/mL and 500 μg/mL in virus infected cultivars (Fig 4).

## Discussion

We detected and identified GFLV in two grape cultivars (Siahe zarqan and Fars) with viral symptom. Specific bands of RT-PCR product were observed at the position corresponding to the excepted size of DNA amplification products of about 118 bp for GFLV. GFLV is one of the virus diseases in grape worldwide.It causes wide leaf yellowing, and fruit deformation. It reduced fruit quality and shortening the lifespan of infected plants in the vineyard (14).

Previous studies on some Iranian grape cultivars have shown that, the incidence of grapevine viruses was found to be low with average values of 11.1% for GFLV, 8.6% for GFkV, 8.4% for GVA, 6.6% for ArMV, 6.4% for GLRaV-3, 2.8% for RpRSV and 0.35% for VTRs (5). In this study, GFLV infected and healthy cultivars were tested for cytotoxicity against MDA-MB-231and HEK 293; although, our results confirmed the existence of GFLV in Iranian vineyards.

All tested extracts showed a range of limited to moderate activity. Previous studies on some white and red cultivars of *V. vinifera* have shown that the leaves and fruits of the grape possess inhibitory activity against various cancers, including colon, esophagus, lung, liver, mammary and skin cancers (14). In a study on mice, grapes have been shown to possess excellent anticancer properties (15). Gurbuz et al. (16) reported that grapes contain phenolic compounds, including resveratrol, flavon-3-ols, caffeic acid, proanthocyanidins and quercetin, which have anticancer property. In the present study, methanolic extracts of skin from red cultivars showed the most cytotoxicity against MDA-MB-231 cell compared to methanolic extracts of leaf, pulp and seed. Red grapes have been reported to be rich sources of proanthocyanidins and anthocyanidins (17). A good correlation has been shown between the proanthocyanidin and anthocyanidin contents in skins and seeds of red grape varieties and anticancer activity (18).

It has been observed that proanthocyanidins in grapes has significant dose-dependent inhibition of the proliferation and viability of the cultured breast cancer cells. In addition, proanthocyanidins in grapes reveal the induction of apoptosis involved both caspase activation-dependent and activation-independent pathways (19). The results showed that cytotoxic activity in extracts of young leaves in red and white grape varieties were lower than that in extracts of the old leaves. It may be due to the higher levels of polyphenols in old leaves. Our findings confirmed by Perez and Gonzalez (20) which reported late harvest of grapes results in an increase in the polyphenol content.

In this study, the results of cytotoxic activities of GFLV infected cultivars were found to be more efficient than uninfected cultivars against MDAMB- 231 and HEK 293 cell lines. It is possibly due to the presence of higher levels of polyphenols in the infected leaves. Kumar (21) and Suresh et al. (22) reported that there are higher amounts of total polyphenols in virus infected plants. Overall, the results indicate the cytotoxic activity in extracts of different parts of the plant, and it also revealed that GFLV can potentially increase the cytotoxicity of grape cultivars.

## Conclusion

Methanolic extracts of skin of red cultivars and extracts of old leave of both red and white cultivars may be potentially applied as anticancer agents. In addition, GFLV potentially increases the cytotoxicity effect of grape cultivars.
